# CaNO and eCO Might Be Potential Non-Invasive Biomarkers for Disease Severity and Exacerbations in Interstitial Lung Disease

**DOI:** 10.3390/jcm14238469

**Published:** 2025-11-28

**Authors:** Yuling Zhang, Faping Wang, Min Zhu, Yali Zhang, Linrui Xu, Liangyuan Li, Ping Li, Qibing Xie, Xiaoyan Lv, Jianqun Yu, Yuben Moodley, Huajing Wan, Hui Mao, Fengming Luo

**Affiliations:** 1 Department of Pulmonary and Critical Care Medicine, West China Hospital, Sichuan University, Chengdu 610041, China; 2 Laboratory of Pulmonary Immunology and Inflammation, Frontiers Science Center for Disease-Related Molecular Network, West China Hospital, Sichuan University, Chengdu 610200, China; 3 The Integrated Care Management Center, West China Hospital, Sichuan University, Chengdu 610041, China; 4 Department of Rheumatology and Immunology, West China Hospital, Sichuan University, Chengdu, 610041, China; 5 Department of Dermatology, West China Hospital, Sichuan University, Chengdu 610041, China; 6 Department of Radiology, West China Hospital, Sichuan University, Chengdu 610041, China; 7 NHMRC Centre of Research Excellence in Pulmonary Fibrosis, Camperdown, NSW 2050, Australia; 8 Centre for Respiratory Health, School of Biomedical Sciences, University of Western Australia, Nedlands, WA 6009, Australia; 9 Cell Biology Group, Institute for Respiratory Health, Nedlands, WA 6009, Australia; 10 Department of Respiratory Medicine, Fiona Stanley Hospital, Murdoch, WA 6150, Australia

**Keywords:** fractional exhaled nitric oxide testing, interstitial lung disease, respiratory function tests, prognosis

## Abstract

**Background:** Interstitial lung diseases (ILDs) often progress quickly and are associated with a poor prognosis. New noninvasive biomarkers to assist in the classification and prognostication of ILD are needed. Exhaled nitric oxide (FeNO), Cavity nitric oxide (CaNO), and carbon monoxide (eCO) are biomarkers of airway inflammation, widely used in respiratory inflammatory diseases such as asthma and chronic obstructive pulmonary disease (COPD). However, their value in ILD remains unclear. **Objective**: To evaluate the potential diagnostic and prognostic value of FeNO, CaNO, and eCO in ILD, and explore their integration into clinical practice. **Methods:** A total of 237 patients were recruited for the study, including 14 with idiopathic pulmonary fibrosis (IPF), 46 with interstitial pneumonia with autoimmune features (IPAF), 19 with mixed connective tissue disease–associated ILD (MCTD-ILD), 65 with polymyositis/dermatomyositis-associated ILD (PM/DM-ILD), 17 with rheumatoid arthritis-associated ILD (RA-ILD), 7 with systemic lupus erythematosus-associated ILD (SLE-ILD), 19 with Sjögren’s syndrome-associated ILD (SS-ILD), and 50 with systemic sclerosis-associated ILD (SSc-ILD). Multiple-flow FeNO and eCO analyses were performed in this population. The associations of these biomarkers with pulmonary function, acute exacerbations, and radiologic fibrosis classification were evaluated. **Results:** Patients with IPF exhibited significantly higher levels of FeNO at 50 mL/s (FeNO50) compared to those with connective tissue disease-associated ILD (CTD-ILD) and IPAF. Both CaNO and eCO were negatively correlated with pulmonary function parameters, particularly forced vital capacity (FVC) and diffusing capacity of the lung for carbon monoxide (DLCO). Receiver operating characteristic (ROC) curve analysis indicated that CaNO is a reliable biomarker for acute exacerbation, with an area under the ROC curve (AUC) of 0.8887, and a cutoff value of 6.35. Additionally, CaNO > 6.35 was associated with a relative risk (RR) of 12.87 for acute exacerbation (AE) compared to CaNO ≤ 6.35. Moreover, both CaNO and eCO levels were significantly higher in the fibrotic ILD group compared to the non-fibrotic group, with ROC analysis indicating AUCs of 0.7173 for CaNO and 0.6875 for eCO. **Conclusions:** FeNO, CaNO, and eCO can provide strong support for the early diagnosis and monitoring of ILD, especially with CaNO playing a crucial role in predicting acute exacerbations. Integrating these biomarkers into clinical practice can help doctors more accurately assess the progression of ILD and develop personalized treatment plans, ultimately improving the prognosis of ILD patients. Future research is needed to validate the effectiveness of these biomarkers in clinical management, facilitating their integration as standard tools for clinical monitoring.

## 1. Introduction

Interstitial lung disease (ILD) is a heterogeneous group of disorders characterized by inflammation and/or fibrosis of the lung parenchyma, ultimately leading to irreversible pulmonary fibrosis. The global disease burden has been steadily increasing over the years [1,2]. Epidemiological studies report that the incidence of ILD ranges from 1 to 31.5 cases per 100,000 person-years, while prevalence estimates range from 6.3 to 71 per 100,000 people, indicating its significant public health impact [3].

In China, the most common subtype of ILDs was idiopathic pulmonary fibrosis (IPF), followed by connective tissue disease associated ILD (CTD-ILD) and interstitial pneumonia with autoimmune features (IPAF), with a predominance of male patients [4,5]. CTD-ILD includes rheumatoid arthritis-associated ILD (RA-ILD), Sjögren’s syndrome-associated ILD (SS-ILD), systemic sclerosis-associated ILD (SSc-ILD), mixed connective tissue disease-associated ILD (MCTD-ILD), polymyositis/dermatomyositis-associated ILD (PM/DM-ILD) and systemic lupus erythematosus-associated ILD (SLE-ILD) [6,7]. Despite advances in clinical practice, the current monitoring approach to ILD—mainly based on high-resolution computed tomography (HRCT), pulmonary function testing, and serological assessment—still has important limitations [8,9,10]. These methods may involve radiation exposure, high costs, and limited sensitivity for early-stage disease. In recent years, there has been significant research into non-invasive diagnostic methods, such as the application of ultrasound in SSc-ILD [11]. We hope to identify new non-invasive biomarkers for the early diagnosis and monitoring of ILD.

Exhaled gas analysis, such as exhaled nitric oxide (FeNO) and exhaled carbon monoxide (eCO), is non-invasive and easily accessible, making it widely used for assessing airway inflammation in diseases like asthma and chronic obstructive pulmonary disease (COPD) [12,13].

eCO levels are positively correlated with exposure to second-hand smoke and biomass/coal combustion. Elevated eCO levels (≥7 ppm) are strongly associated with airflow limitation and the prevalence of related respiratory symptoms. Higher eCO concentrations are also linked to lower forced expiratory volume in 1 s (FEV_1_%) and accelerated lung function decline [14,15,16].

Nitric oxide (NO), which is produced by various cell types in the respiratory tract, plays a pivotal role in the fibrosis process [17]. Specifically, exhaled NO is measured at different flow rates to assess airway and alveolar inflammation.

FeNO50, measured at a flow rate of 50 mL/s, is commonly used in clinical practice to assess upper airway inflammation, particularly in monitoring allergic airway reactions and asthma [18,19]. FeNO200, measured at a flow rate of 200 mL/s, is used to evaluate NO concentrations in the more distal airways such as the trachea. It reflects deeper airway inflammation and is often used to differentiate more severe airway diseases or for research purposes. CaNO represents the concentration of NO in the airway cavities and is indicative of inflammation in the deeper parts of the airways, including the alveoli. It can help assess the severity of pulmonary diseases, particularly when assessing alveolar–capillary membrane diffusion and damage to the airway-alveolar interface [20].

Previous studies have shown that CO-Hb levels are associated with the severity of ILD [21], but the relationship between eCO and ILD has been rarely reported. Additionally, the literature on FeNO in ILD is limited, with most studies involving small sample sizes and primarily comparing IPF or ILD with healthy controls [22]. However, existing studies lack comparative analysis across different ILD subtypes, and no studies have yet explored whether these biomarkers are associated with acute exacerbations or radiological fibrosis classification of ILD. Based on the available evidence, we hypothesize that both FeNO and eCO can serve as reliable non-invasive biomarkers for the early detection and prognosis of ILD, and that elevated levels of these biomarkers may correlate with disease severity.

## 2. Methods

### 2.1. Study Design and Subjects

This prospective observational study followed a cohort of ILD patients who visited West China Hospital of Sichuan University between September 2019 and September 2024, with a limited duration of longitudinal follow-up. This study combines the analysis of exhaled gas data, pulmonary function tests, and imaging results, with follow-up for up to four years. Data collection involved multiple assessments at various time points. For the majority of patients, exhaled gas data and pulmonary function tests were performed synchronously, on the same day. However, for other patients, measurements were taken at different follow-up visits, with a gap of 1 to 3 months between assessments. The time window between exhaled gas testing and chest CT scans was typically within 3 months, ensuring proper synchronization of the data. This approach facilitated a comprehensive evaluation of disease progression over time. This study was conducted in compliance with the Declaration of Helsinki, as revised in 1983. It was approved by the Ethics Committee on Biomedical Research at West China Hospital of Sichuan University (approval number 2019–246). Written informed consent was obtained from all participants in the prospective portion of the study.

The inclusion criteria were as follows: 1. Age between 18 and 80 years, with no gender restriction; 2. Diagnosed with a form of interstitial lung disease (according to the ATS/ERS 2018 guidelines [23], including but not limited to IPF, IPAF, and CTD-ILD; 3. Able to complete FeNO and eCO measurements and pulmonary function assessments according to standard protocols; 4. Complete clinical and imaging records; 5. Signed informed consent. Exclusion criteria included: 1. Co-existing asthma, COPD, or other chronic respiratory diseases that could affect FeNO or eCO values; 2. Current smokers; 3. History of active respiratory infection or other acute respiratory diseases within the past month; 4. Patients unable to cooperate with the required tests.

The diagnostic criteria for acute exacerbation of interstitial lung disease (AE-ILD) were based on the international working group report by Collard et al. [24]: 1. A prior or current diagnosis of ILD; 2. Unexplained worsening of dyspnea or the onset of new dyspnea, typically lasting less than 1 month, and cannot be fully explained by heart failure or fluid overload; 3. HRCT showing new bilateral ground-glass opacities or consolidation; 4. Triggers include infection, gastroesophageal reflux, microaspiration, surgery, bronchoscopy, etc.

**Figure 1:**
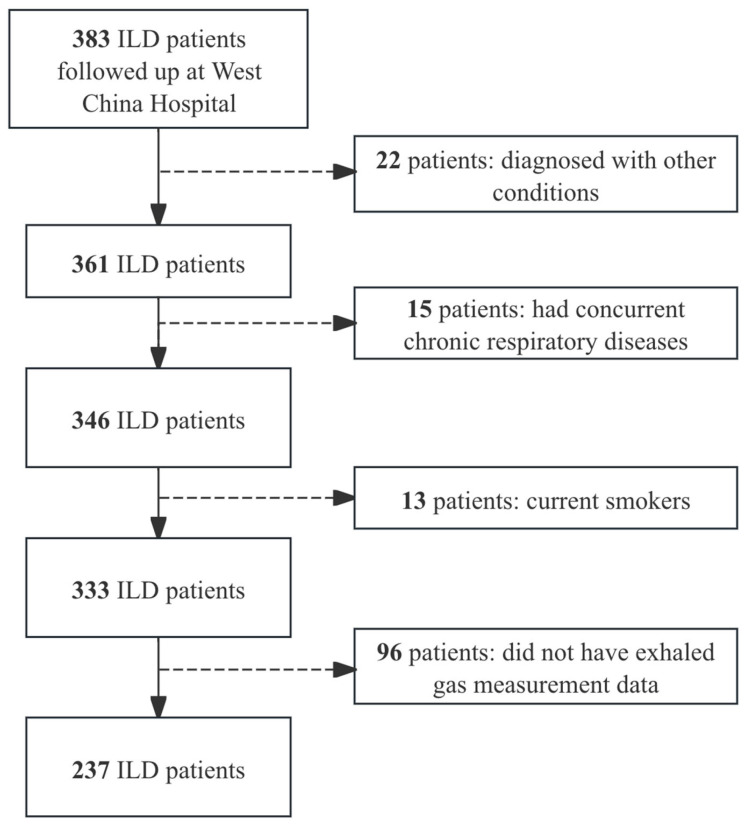
Patient enrollment flowchart. Solid arrows indicate patients who met the inclusion criteria and were included in the cohort, while dashed arrows represent patients who were excluded based on specific criteria.

The patient enrollment flowchart is shown in Figure 1. A total of 237 patients were included, with 14 cases of IPF, 46 cases of IPAF, 19 cases of MCTD-ILD, 65 cases of PM/DM-ILD, 17 cases of RA-ILD, 7 cases of SLE-ILD, 19 cases of SS-ILD, and 50 cases of SSc-ILD.

### 2.2. Exhaled Gas Measurement

In this study, the Sunvou-CA2122 exhaled gas analyzer (Wuxi Sunvo Medical Electronics Co., Ltd., Wuxi, China) was used to measure the concentrations of nitric oxide (NO) and carbon monoxide (CO) in exhaled breath. The device supports three sampling modes: online sampling, offline sampling, and tidal breath sampling. Each test required a sample volume of 30 mL of exhaled air, with exhalation flow rates for mouth exhalation set to 50 ± 10% mL/s and 200 ± 10% mL/s, while the nasal exhalation flow rate was set at 10 mL/s. These settings allowed for segmented testing of NO concentrations in the bronchial, alveolar, and nasal regions. The analyzer supports the simultaneous measurement of multiple parameters, including FeNO50, FeNO200, CaNO, combined bronchial and alveolar NO testing (FeNO50 + FeNO200 + CaNO). The collection of exhaled gas followed the recommendations of the ATS/ERS 2011 guidelines for interpreting FeNO for clinical applications [25], and the ERS technical standards for exhaled biomarkers in lung diseases [26]. The inhalation filter, which contains potassium permanganate, activated charcoal, and N99 fiber layers, effectively filters harmful gases, including bacteria, viruses, and other microorganisms, preventing cross-contamination. The detection range for NO is 0 ppb to 3000 ppb, and for CO, it is 0 ppm to 250 ppm. The accuracy for NO is <±3 ppb for concentrations below 50 ppb and <±10% for concentrations above 50 ppb, while the accuracy for CO is <±2 ppm for concentrations below 20 ppm and <±10% for concentrations above 20 ppm. The device can detect 16 common exhaled molecules, including NO_2_, with an interference level below 3 ppb. The relative deviation (CV) for reproducibility should be ≤10%, and the relative drift during measurements within 2 h (concentration change rate) should be ≤±10%. All data were collected using the provided PC-based software, which automatically generates test reports and allows for the retrieval and review of historical data. The collected data were integrated into the hospital’s Health Information System (HIS) for seamless record-keeping and analysis.

### 2.3. High-Resolution Chest CT Examination

All patients underwent HRCT before being included in the study. Two senior experts independently classified each HRCT as fibrotic or non-fibrotic, inter-observer reliability for HRCT images was assessed using Cohen’s kappa statistic. A scan was deemed fibrotic if it showed any of the following hallmark features: reticular abnormalities, traction bronchiectasis/bronchiolectasis, or honeycombing. These characteristics are commonly observed in diseases such as IPF and reflect advanced fibrotic changes [27]. Scans were labeled as non-fibrotic when no fibrotic signs were present, and interstitial abnormalities were limited to ground-glass opacities (GGO), subpleural reticulation, and/or centrilobular nodules, without tractional changes, honeycombing, or architectural distortion. These imaging features indicate that the inflammatory process has not yet progressed to significant fibrosis [27,28]. These imaging distinctions are crucial for differentiating between fibrotic and non-fibrotic ILD, as well as for assessing disease progression and formulating treatment strategies.

### 2.4. Pulmonary Function Test

Pulmonary function tests were performed according to ERS standards [29]. The parameters assessed included forced expiratory volume in 1 s (FEV_1_), forced vital capacity (FVC), total lung capacity (TLC), residual volume (RV), and diffusing capacity for carbon monoxide (DLCO). The FEV_1_/FVC ratio was calculated, and all values were expressed as percentages of the predicted reference values to ensure standardization and comparability.

### 2.5. Data Processing and Analysis

All statistical analyses were performed using SPSS 25.0 software. Descriptive statistics were used for baseline data, with categorical data presented as absolute numbers and relative frequencies. Continuous data were expressed as means with standard deviations. A power analysis was conducted using G*Power to determine the adequacy of the sample size.

For the analysis of exhaled gas values across different disease groups, one-way analysis of variance (ANOVA) was performed following normality testing, with Bonferroni correction applied to adjust the *p*-values. For the analysis of the correlation between exhaled gas values and pulmonary function, Spearman’s rank correlation was used, as pulmonary function data exhibited a skewed distribution. Differences in exhaled gas values between AE-ILD and no AE-ILD groups, as well as between fibrotic and non-fibrotic ILD groups, were analyzed using independent samples *t*-tests, as the data were normally distributed. Additionally, receiver operating characteristic (ROC) curve analysis was used to evaluate the diagnostic performance of CaNO and eCO for identifying patients with AE-ILD or fibrotic ILD. The area under the curve (AUC) was calculated to assess the sensitivity and specificity of each biomarker in distinguishing between different disease phases and types. A *p*-value of <0.05 was considered statistically significant. All results were assessed using GraphPad Prism 6.0 software.

Multivariate analysis, including linear or logistic regression models, was used to adjust for potential confounding factors such as age, sex, smoking history, and treatment regimens. Additionally, due to the large number of ILD subtypes included in the cohort and the small sample sizes in some subgroups, we did not perform subgroup comparisons. Instead, we used multivariate adjustment to ensure that the observed associations between biomarkers and disease parameters were not confounded by these variables.

During patient inclusion, we excluded individuals without complete clinical data. However, some patients had missing data for certain exhaled gas or pulmonary function parameters, for example, having FeNO50 data but lacking CaNO data. To handle this, we adopted an analysis strategy based on available data. Specifically, when analyzing tthe relationship between CaNO and pulmonary function, we included only patients with the corresponding data to ensure the completeness and accuracy of the analysis. Similarly, other analyses were conducted using the corresponding available data. While this approach addresses missing data to some extent, we acknowledge that it may limit the generalizability of certain conclusions. Therefore, future studies should consider more systematic methods for handling missing data, such as multiple imputation, to enhance the stability and reliability of the results.

## 3. Results

### 3.1. Baseline Characteristics of Population

The demographic characteristics of the study groups are summarized in Table 1. The IPF group (*n* = 14) had significant differences compared to other disease groups in terms of age, gender, and smoking history. The average age of the IPF group was 68.57 ± 6.34 years, and 12 of the 14 patients had a history of smoking. Adjustment for these factors was performed in subsequent analyses, as detailed in the Section 2.

### 3.2. FeNO50 Is Significant High in IPF Patients

**Figure 2:**
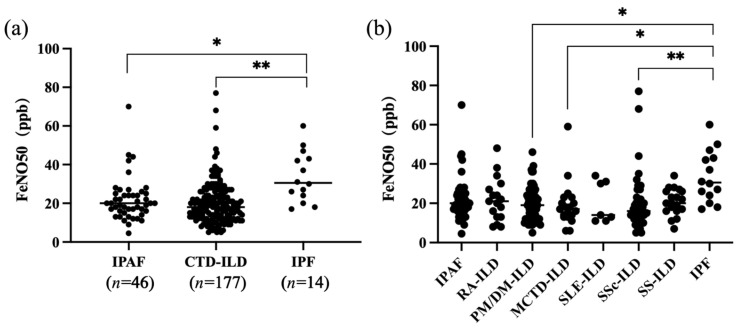
Comparison of FeNO50 values between different subgroups. (**a**) FeNO50 values in the IPF, CTD-ILD, and IPAF groups. (**b**) FeNO50 values in the IPF and IPAF groups versus other CTD-ILD subgroups. Including MCTD-ILD (*n* = 19), PM/DM-ILD (*n* = 65), RA-ILD (*n* = 17), SLE-ILD (*n* = 7), SS-ILD (*n* = 19), and SSc-ILD (*n* = 50). “*” represents a *p*-value < 0.05 and “**” represents a *p*-value < 0.01.

We compared the distribution of four exhaled gas values across different disease groups (Figure 2). The results showed that FeNO50 levels were significantly higher in the IPF group compared to both the CTD-ILD (*p* = 0.0024, 95% CI for the mean difference = [1.83, 6.18], Bonferroni-corrected *p*-value = 0.0061) and IPAF (*p* = 0.0357, 95% CI for the mean difference = [20.03, 23.27], Bonferroni-corrected *p*-value = 0.0324) groups. The overall effect size measured by Cohen’s f was 0.31, indicating a moderate effect across the groups. Within the CTD-ILD subgroups, FeNO50 levels were significantly higher in the IPF group compared to SSc-ILD (*p* = 0.0075, 95% CI for the mean difference = [3.966, 31.32], Bonferroni-corrected *p*-value = 0.0071), MCTD-ILD (*p* = 0.0481, 95% CI for the mean difference = [4.721, 32.38], Bonferroni-corrected *p*-value = 0.0461) and PM/DM-ILD (*p* = 0.0456, 95% CI for the mean difference = [4.366, 30.65], Bonferroni-corrected *p*-value = 0.0463). The overall effect size measured by Cohen’s f was 0.26, indicating a moderate effect across the groups. However, no significant differences were found in FeNO200, eCO, or CaNO levels among those disease groups.

### 3.3. Association of Exhaled Gas Levels with Pulmonary Function

**Figure 3:**
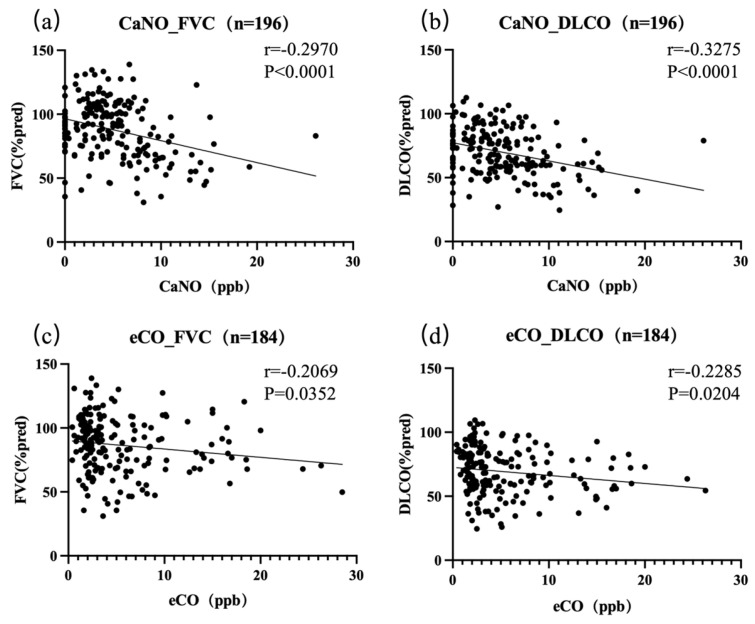
Correlation between CaNO, eCO, and pulmonary function in ILD patients. (**a**) CaNO levels are negatively correlated with FVC (forced vital capacity percentage). (**b**) CaNO levels are negatively correlated with DLCO (diffusing capacity for carbon monoxide percentage). (**c**) eCO levels are negatively correlated with FVC. (**d**) eCO levels are negatively correlated with DLCO.

Due to missing exhaled gas and pulmonary function data in some patients, a total of 196 patients were included in the analysis of the correlation between CaNO and pulmonary function, while 184 patients were included in the eCO analysis. The results showed that both CaNO and eCO were negatively correlated with FVC % predicted and DLCO % predicted (Figure 3). Specifically, CaNO showed a significant negative correlation with FVC % predicted (r = −0.2970, 95% CI = [−0.4231, −0.1596], R^2^ = 0.1040, *p* < 0.0001) and DLCO % predicted (r = −0.3275, 95% CI = [−0.4508, −0.1920], R^2^ = 0.1059, *p* < 0.0001). Additionally, eCO was negatively correlated with FVC % predicted (r = −0.2069, 95% CI = [−0.3455, −0.05942], R^2^ = 0.02426, p = 0.0352), and DLCO % predicted (r = −0.2285, 95% CI = [−0.3656 to −0.08156], R^2^ = 0.02950, *p* = 0.0204). These findings suggest that higher levels of CaNO and eCO are associated with reduced pulmonary function, particularly in terms of FVC and DLCO.

### 3.4. Exhaled Biomarkers as Indicators of Fibrotic ILD and Acute Exacerbation

**Figure 4:**
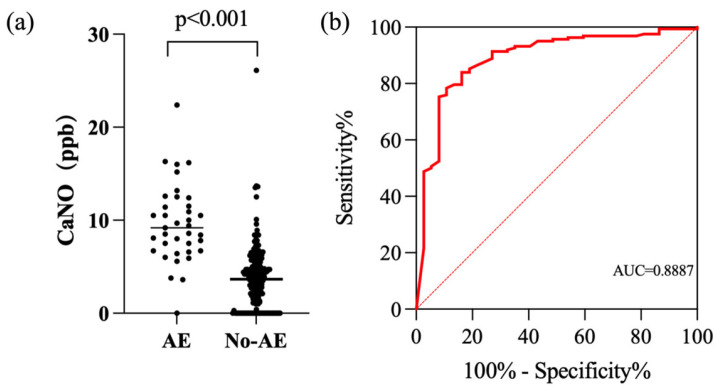
Correlation between CaNO and acute exacerbation. (**a**) Comparison of CaNO levels between patients with and without acute exacerbation (*p* < 0.001). (**b**) ROC curve for CaNO in distinguishing acute exacerbation (AUC = 0.8887).

We compared all exhaled gas data (including FeNO50, FeNO200, CaNO, and eCO) with the occurrence of acute exacerbation (AE) (Figure 4). Due to missing CaNO data in some patients, a total of 199 patients were included in the analysis of the relationship between CaNO and acute exacerbation, of whom 37 patients experienced acute exacerbations within one year of inclusion in the follow-up. The analysis revealed that the CaNO levels were significantly higher in patients who experienced AE within one year after enrollment compared to those who did not. ROC curve analysis revealed an area under the curve (AUC) of 0.8887 (95% CI: [0.8267, 0.9508]), with a cutoff value of 6.35, yielding a sensitivity of 83.8% and specificity of 84%. For individuals with CaNO > 6.35, the relative risk (RR) of acute exacerbation was 12.87 times (95% CI: [4.88, 33.94]) higher compared to those with CaNO ≤ 6.35, indicating a strong association between CaNO levels and the occurrence of acute exacerbations.

**Figure 5:**
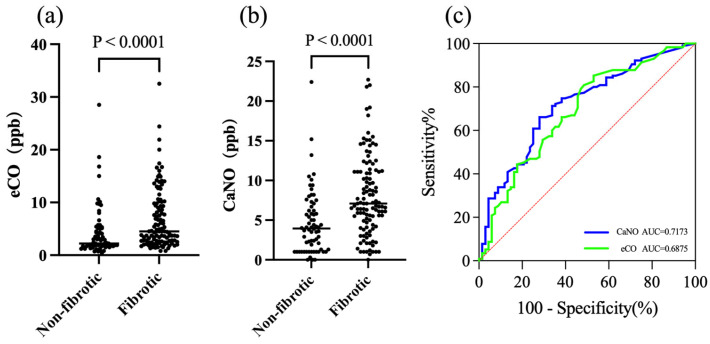
Correlation between CaNO, eCO and fibrotic ILD. (**a**,**b**) Comparison of eCO and CaNO levels between fibrotic and non-fibrotic ILD (*p* < 0.0001). (**c**) ROC curve comparison of eCO and CaNO for fibrosis (AUC: CaNO = 0.7173, eCO = 0.6875).

Moreover, we compared all exhaled gas data with fibrosis classification and found both CaNO and eCO were significantly associated with fibrosis classification, with higher levels in the fibrotic group compared to the non-fibrotic group (Figure 5). Due to a time gap between CT scans and exhaled gas collection in some patients, only 183 patients with concurrent imaging data were included in the HRCT fibrosis classification analysis, with 115 patients diagnosed with fibrotic ILD and 68 patients diagnosed with non-fibrotic ILD. The Cohen’s kappa for inter-observer reliability in HRCT image assessment was calculated to be 0.87, indicating a high level of agreement between the two senior experts. Both eCO and CaNO levels were significantly higher in the fibrotic ILD group compared to the non-fibrotic ILD group (*p* < 0.0001). To assess the diagnostic performance of these biomarkers, we performed a ROC curve analysis. The area under the ROC curve (AUC) for CaNO was 0.7173 (95% CI: [0.6412, 0.7933]), while for eCO, it was 0.6875 (95% CI: [0.6069, 0.7681]). Furthermore, we determined optimal cut-off values for both biomarkers: eCO at 2.25 ppb with a sensitivity of 81.9% and specificity of 51.5%, and CaNO at 5.85 ppb with a sensitivity of 67.8% and specificity of 70.6%. The DeLong test comparing their AUC values for diagnosing fibrotic ILD revealed no significant difference (*p* > 0.05). This suggests that both biomarkers provide similar diagnostic performance in distinguishing fibrotic from non-fibrotic ILD. Additionally, the combined diagnostic performance of CaNO and eCO was not significantly different from the individual diagnostic performance of each biomarker.

## 4. Discussion

In this study, we evaluated exhaled nitric oxide parameters (FeNO50, FeNO200, CaNO) and eCO as potential non-invasive biomarkers in various subtypes of ILD. We observed that FeNO50 levels were significantly higher in patients with IPF compared to those with IPAF and CTD-ILD. Additionally, we found that CaNO and eCO were significantly associated with fibrotic HRCT patterns and pulmonary function impairment. Notably, CaNO levels were markedly higher in patients who experienced AE-ILD within one year, with an AUC of 0.8887.

Given that no healthy controls were enrolled, we interpreted our results against published ranges obtained under comparable measurement conditions. In non-smoking healthy adults, FeNO 50 is typically ~10–25 ppb. For FeNO200, a universal “healthy cutoff” has not been established, but values are generally lower than FeNO50 [30]. CaNO in healthy adults is usually ~2–4 ppb [31]. For eCO, non-smokers commonly show ~1–4 ppm, and many studies use ≥7 ppm to indicate active smoking or abnormal exposure [32].

Previous studies have primarily focused on comparing FeNO levels between patients with ILD and healthy controls [33,34]. Some studies have also compared FeNO differences across various ILD subtypes. For instance, Guilleminault et al. found that FeNO50 levels were significantly higher in patients with hypersensitivity pneumonitis (HP) compared to those with IPF, CTD-ILD, or drug-induced ILD [35]. This is likely due to the strong association between FeNO and allergic diseases [36]. Similarly, Cameli et al. discovered that FeNO50 levels were higher in IPF patients compared to both healthy controls (HC) and sarcoidosis patients [37,38]. However, to our knowledge, direct comparisons of FeNO50 between IPF and either CTD-ILD or IPAF have not been reported. In our cohort, FeNO50 was significantly higher in IPF than in CTD-ILD and IPAF, thereby extending prior evidence by demonstrating subtype-specific differences beyond the previously reported contrasts. The observed increase in FeNO50 in IPF may be driven by both structural and treatment-related factors. Pathologically, IPF is characterized by bronchiolization of distal airspaces, whereby fibrotic “honeycomb” cysts are frequently lined by airway-type mucociliary epithelium—including p63^+^/KRT5^+^ basal cells, ciliated cells, and MUC5B-producing goblet cells—rather than by normal alveolar epithelium [39]. The expansion of goblet and basal cell populations, which can express inducible nitric oxide synthase (iNOS) under inflammatory conditions, provides a plausible source of additional local nitric oxide within the remodeled distal lung. Clinically, CTD-ILD/IPAF are more often treated with systemic corticosteroids and other immunosuppressants that suppress airway iNOS and lower FeNO [40,41,42], whereas IPF is typically managed with antifibrotics without maintenance immunosuppression. Thus, for comparable disease burden, FeNO50 tends to be lower in CTD-ILD/IPAF, yielding a higher FeNO50 level in IPF.

In our cohort, higher CaNO tracked worse physiology and more advanced structural disease. CaNO was correlated inversely with DLCO%Pred and FVC%Pred and was significantly higher in fibrotic versus non-fibrotic HRCT patterns, which is consistent with previous studies [37,43,44]. Evidence suggests that higher CaNO reflects ongoing alveolar inflammation and nitrosative stress accompanied by alveolar–capillary membrane thickening [45], which reduces NO clearance and compromises gas transfer, thereby leading to lower DLCO and FVC. In fibrotic ILD, inflammatory cells—particularly alveolar macrophages—overexpress iNOS, leading to increased local NO production [22]. NO then upregulates VEGF via the PI3K/Akt pathway, amplifying endothelial activation, permeability, and fibroproliferative remodeling [17], together with impaired NO washout across a thickened alveolar–capillary membrane, this raises CaNO and increases exhaled NO.

Additionally, baseline CaNO also stratified short-term risk—patients who developed AE-ILD within one year had markedly higher CaNO at enrollment. AE-ILD is commonly triggered by infections, with imaging showing an increase in ground-glass opacities or consolidation [46,47]. CaNO, which reflects alveolar inflammation, has been found to correlate with the severity of pulmonary inflammation. Elevated CaNO levels indicate ongoing alveolar inflammation, which may be a critical factor in the pathogenesis of acute exacerbations. As mentioned earlier in this article, persistent alveolar inflammation, along with impairment of the alveolar–capillary diffusion barrier, creates an unstable alveolar microenvironment that lowers the threshold for acute injury. This could also explain the higher exhaled nitric oxide observed in patients who experience acute exacerbations. Notably, the AUC was greater than 0.8, indicating that CaNO could potentially be used to predict the occurrence of AE-ILD within one year in ILD patients. This finding carries significant clinical implications, as it may help identify patients at high risk for acute exacerbations, thereby enabling timely interventions.

eCO was also associated with functional impairment and fibrotic ILD, and negatively correlated with FVC%Pred and DLCO%Pred and was higher in fibrotic than non-fibrotic ILD. Research on eCO in ILD remains limited. However, studies targeting other diseases have shown that eCO is inversely correlated with pulmonary function parameters [48,49]. In parallel, eCO—an HO-1–derived product—captures oxidative stress and macrophage activation, its increase is biologically consistent with greater loss of ventilated lung units, providing a rationale for its negative relationship with spirometric and diffusing capacity measures [50,51]. In fibrotic ILD, HO-1 plays a key role in promoting IL-10 production [52,53]. IL-10, in turn, promotes the differentiation of M2 macrophages, which are known to contribute to lung fibrosis [54]. The increased number of M2 macrophages enhances fibrosis by stimulating fibroblast proliferation and collagen deposition in the lung. IL-10 also upregulates HO-1 expression, creating a positive feedback loop that further amplifies inflammation and fibrosis [55]. Since CO is a product of HO-1, the elevated eCO levels observed in fibrotic ILD patients may reflect this intensified IL-10-HO-1 interaction and the ongoing fibrotic processes. This feedback loop between HO-1 and IL-10, alongside CO production, may help explain the higher eCO levels in fibrotic ILD compared to non-fibrotic ILD patients.

Taken together, CaNO and eCO quantify complementary facets of distal inflammatory/oxidative injury that co-vary with physiologic decline, with CaNO additionally demonstrating prognostic utility for AE-ILD.

Although all these correlations are statistically significant, the strength of the relationship between exhaled gases and pulmonary function ranges from weak to moderate, with eCO and CaNO showing moderate sensitivity and specificity for diagnosing fibrotic ILD. In this study, we controlled for basic factors such as age, sex, and smoking status to minimize potential confounding effects. However, environmental factors such as air pollution and occupational exposure may also influence lung function and radiographic findings, potentially interacting with the biomarkers under study.

Nevertheless, eCO and CaNO as non-invasive biomarkers still have significant potential clinical applications. They hold significant potential for early screening and prognosis assessment, particularly in high-risk patients, such as the elderly and those with environmental or occupational exposures. Through these biomarkers, clinicians can identify potential poor prognosis patients at early stages of the disease, providing more opportunities for intervention and treatment. Additionally, the non-invasive nature of these biomarkers makes them especially suitable for clinical practice, particularly in patient populations that require frequent monitoring.

However, in this study, no significant differences in exhaled gas values were found between certain ILD subtypes. From a pathophysiological perspective, NO and CO reflect not only local production but also the alveolar–capillary diffusion and clearance processes. In ILD, microvascular remodeling and capillary rarefaction can impair NO/CO clearance into the bloodstream, while oxidative stress may increase their production. This process may lead to elevated CaNO/eCO levels in fibrotic ILD and acute exacerbations, independently of airway inflammation. This hypothesis helps explain why some ILD subtypes show modest or absent differences in biomarker levels.

However, this study provides valuable data, but there are still some limitations. First, despite conducting a long-term follow-up of the study cohort, the sample size for rare ILD subtypes such as IPF was relatively small. Furthermore, the IPF group was entirely male, consistent with studies showing higher prevalence in men. This gender imbalance may limit the generalizability of our findings. This issue is due to our study being single-center, and multi-center studies with larger, more diverse cohorts are needed. Second, potential confounding factors, such as environmental CO exposure and occupational exposures, were not fully controlled in this study, and these factors may influence exhaled gas levels. Third, the study did not include healthy controls, and there was no data on the BMI of the participants, and interpretation relied on published reference ranges obtained under comparable measurement conditions. Fourth, short-interval test–retest reliability was not assessed as follow-ups occurred every 3–6 months. Fifth, although HRCT scans were classified as fibrotic or non-fibrotic, granular image–biomarker correlations (e.g., extent of ground-glass opacities, traction bronchiectasis, honeycombing) and standardized CT-based AE severity grading were not assessed. An additional limitation of this study is that the exacerbation analysis was restricted to a one-year follow-up period, which may not fully capture the long-term prognostic value of the biomarkers. The relatively short duration of follow-up restricts our ability to assess the biomarkers’ predictive capacity for longitudinal disease progression and chronic exacerbations. Therefore, future research should further control for these confounding variables and explore their impact on biomarker measurements. Finally, while our study provides clinically meaningful findings, additional long-term follow-up data in future prospective studies are needed to validate the role of these biomarkers in the long-term progression of the disease.

## 5. Conclusions

Our study demonstrates that patients with IPF exhibited significantly higher levels of FeNO50 compared to those with CTD-ILD and IPAF, which may help in distinguishing ILD subtypes. CaNO and eCO are promising non-invasive biomarkers for evaluating lung function impairment and distinguishing fibrotic types in patients with ILD. Elevated CaNO was particularly associated with both fibrotic HRCT patterns and acute exacerbations, suggesting its potential as a predictive marker for disease flare-ups. Exhaled gas analysis, as a non-invasive tool, plays a crucial role in monitoring ILD progression.

This study provides novel insights into the role of exhaled biomarkers, such as FeNO and eCO, in the evaluation of interstitial lung disease (ILD). Unlike previous studies that mainly focused on comparing disease populations with healthy controls, our study highlights the differences between ILD subtypes, offering a more comprehensive understanding of how these biomarkers correlate with disease severity and prognosis across various ILD subtypes. This study provides novel insights into the role of exhaled biomarkers, such as FeNO and eCO, in the evaluation of interstitial lung disease (ILD). Unlike previous studies that mainly focused on comparing disease populations with healthy controls, our study highlights the differences between ILD subtypes, offering a more comprehensive understanding of how these biomarkers correlate with disease severity and prognosis across various ILD subtypes. However, further research with larger cohorts and longitudinal follow-ups is required to confirm these biomarkers’ prognostic value and to explore their roles in guiding personalized treatment strategies. Additionally, there is a need for international standardization of CaNO/eCO cutoff values to ensure their widespread use and consistency in clinical practice.

## Figures and Tables

**Table 1:** Demographics, lung function, exhaled gases, and therapy in ILD subgroups.

		IPF (*n* = 14)	IPAF (*n* = 46)	CTD-ILD (*n* = 177)
Demographic Information	Gender (Female/male)	0/14	37/9	155/22
	Age (years)	68.57 ± 6.34	60.28 ± 12.25	53.33 ± 10.85
Medical History	Smoking history (Y/N)	12/2	9/37	23/154
	Occupational/Environmental Exposure (Y/N)	4/10	13/33	38/139
Exhaled Biomarkers	FeNO50 (ppb)	31 ± 13.66	21.86 ± 11.05	20.17 ± 10.68
	FeNO200 (ppb)	13.25 ± 5.71	10.63 ± 5.24	10.06 ± 5.25
	CaNO (ppb)	8.95 ± 6.54	5.52 ± 4.29	5.53 ± 4.32
	eCO (ppb)	5.69 ± 3.92	6.51 ± 6.08	5.59 ± 5.34
Pulmonary Function Measurements	FVC%Pred	82.93 ± 19.16	87.51 ± 24.15	85.16 ± 21.79
	DLCO%Pred	67.54 ± 17.59	68.06 ± 18.40	69.93 ± 18.52
Treatment	Hormone	1 (7.14%)	2 (4.35%)	9 (5.08%)
	Hormone + Immunosuppressive agents	2 (14.29%)	8 (17.39%)	28 (15.82%)
	Hormone + Antifibrotic therapy	1 (7.14%)	14 (30.43%)	18 (10.17%)
	Hormone + Immunosuppressive agents + Antifibrotic therapy	7 (50.00%)	10 (21.74%)	93 (52.54%)
	Antifibrotic therapy	3 (21.43%)	5 (10.87%)	21 (11.86%)
	Antifibrotic therapy + Immunosuppressive agents	0 (0)	7 (15.22%)	8 (4.52%)

Data are presented as n (%) or means± standard deviation (SD). Y: Yes, N: No.

## Data Availability

The data that support the findings of this study are available from the corresponding author upon reasonable request due to privacy and ethical restrictions.

## Abbreviations

AE	acute exacerbation
AE-ILD	acute exacerbation of interstitial lung disease
ANOVA	analysis of variance
AUC	area under the (ROC) curve
CI	confidence interval
COPD	chronic obstructive pulmonary disease
CTD-ILD	connective tissue disease-associated ILD
DLCO	diffusing capacity of the lung for carbon monoxide
eCO	exhaled carbon monoxide
FeNO	fractional exhaled nitric oxide
FeNO50	FeNO at 50 mL/s
FeNO200	FeNO at 200 mL/s
FEV_1_	forced expiratory volume in 1 s
FVC	forced vital capacity
GGO (s)	ground-glass opacity (ies)
HC	healthy controls
HO-1	heme oxygenase-1
HRCT	high-resolution computed tomography
HP	hypersensitivity pneumonitis
IIP	idiopathic interstitial pneumonias
IL-10	interleukin-10
ILD	interstitial lung disease
IPAF	interstitial pneumonia with autoimmune features
IPF	idiopathic pulmonary fibrosis
iNOS	inducible nitric oxide synthase
M2	M2 macrophages
MCTD-ILD	mixed connective tissue disease-associated ILD
NO	nitric oxide
PM/DM-ILD	polymyositis/dermatomyositis-associated ILD
RA-ILD	rheumatoid arthritis-associated ILD
ROC	receiver operating characteristic
RR	relative risk
RV	residual volume
SLE-ILD	systemic lupus erythematosus–associated ILD
SS-ILD	Sjögren’s syndrome-associated ILD
SSc-ILD	systemic sclerosis-associated ILD
TLC	total lung capacity
VEGF	vascular endothelial growth factor
%pred	percent predicted

## References

[1] Zhou M., Zhou Y., Yang X., Zhou K., Zhu X. (2025). Global, regional, and national burden of interstitial lung diseases and pulmonary sarcoidosis from 2000 to 2021: A systematic analysis of incidence, mortality, and disability-adjusted life years. Front. Public Health.

[2] Zhao L., Zhou Y., Jia Y., Wang L., Liu Y., Lv G., Zhang Y., Li J., Ren J., Liu H. (2025). Assessing the global burden of interstitial lung disease and pulmonary sarcoidosis using multiple statistical models: Analysis and future projections. BMC Pulm. Med..

[3] Spagnolo P., Guler S.A., Chaudhuri N., Udwadia Z., Sesé L., Kaul B., Enghelmayer J.I., Valenzuela C., Malhotra A., Ryerson C.J. (2025). Global epidemiology and burden of interstitial lung disease. Lancet Respir. Med..

[4] Guo B., Wang L., Xia S., Mao M., Qian W., Peng X., Zheng Z., Chen R., Han Q., Luo Q. (2020). The interstitial lung disease spectrum under a uniform diagnostic algorithm: A retrospective study of 1945 individuals. J. Thorac. Dis..

[5] Ban C., Yan W., Xie B., Zhu M., Liu Y., Zhang S., Ye Q., Ren Y., Jiang D., Geng J. (2018). Spectrum of interstitial lung disease in China from 2000 to 2012. Eur. Respir. J..

[6] Maher T.M. (2024). Interstitial Lung Disease: A Review. JAMA.

[7] Ryerson C.J., Adegunsoye A., Piciucchi S., Hariri L.P., Khor Y.H., Wijsenbeek M.S., Wells A.U., Sharma A., Cooper W.A., Antoniou K. (2025). Update of the International Multidisciplinary Classification of the Interstitial Pneumonias: An ERS/ATS Statement. Eur. Respir. J..

[8] Szturmowicz M., Garczewska B., Jędrych M.E., Bartoszuk I., Sobiecka M., Tomkowski W., Augustynowicz-Kopeć E. (2019). The value of serum precipitins against specific antigens in patients diagnosed with hypersensitivity pneumonitis—Retrospective study. Cent. Eur. J. Immunol..

[9] Boros P.W., Martusewicz-Boros M.M., Lewandowska K.B. (2025). Assessment of lung function and severity grading in interstitial lung diseases (% predicted versus z-scores) and association with survival: A retrospective cohort study of 6808 patients. PLoS Med..

[10] Raghu G., Remy-Jardin M., Richeldi L., Thomson C.C., Inoue Y., Johkoh T., Kreuter M., Lynch D.A., Maher T.M., Martinez F.J. (2022). Idiopathic Pulmonary Fibrosis (an Update) and Progressive Pulmonary Fibrosis in Adults: An Official ATS/ERS/JRS/ALAT Clinical Practice Guideline. Am. J. Respir. Crit. Care Med..

[11] Hughes M., Bruni C., Cuomo G., Delle Sedie A., Gargani L., Gutierrez M., Lepri G., Ruaro B., Santiago T., Suliman Y. (2020). The role of ultrasound in systemic sclerosis: On the cutting edge to foster clinical and research advancement. J. Scleroderma Relat. Disord..

[12] Ryter S.W., Sethi J.M. (2007). Exhaled carbon monoxide as a biomarker of inflammatory lung disease. J. Breath Res..

[13] Shen Y., Yang L.-L., Ning G.-L., Teng X.-B., Shi J.-F., Cui S.-S., Cao Z.-X., Zhang Y.-B., Han M.-F. (2025). Analysis of exhaled nitric oxide and its influencing factors in patients with chronic obstructive pulmonary disease. Front. Med..

[14] Zhang Q., Li L., Smith M., Guo Y., Whitlock G., Bian Z., Kurmi O., Collins R., Chen J., Lv S. (2013). Exhaled carbon monoxide and its associations with smoking, indoor household air pollution and chronic respiratory diseases among 512,000 Chinese adults. Int. J. Epidemiol..

[15] Fabricius P., Scharling H., Løkke A., Vestbo J., Lange P. (2007). Exhaled CO, a predictor of lung function?. Respir. Med..

[16] Bradicich M., Schuurmans M.M. (2020). Smoking status and second-hand smoke biomarkers in COPD, asthma and healthy controls. ERJ Open Res..

[17] Iyer A.K.V., Ramesh V., Castro C.A., Kaushik V., Kulkarni Y.M., Wright C.A., Venkatadri R., Rojanasakul Y., Azad N. (2015). Nitric oxide mediates bleomycin-induced angiogenesis and pulmonary fibrosis via regulation of VEGF. J. Cell. Biochem..

[18] Zeng G.-S., Chen H., Chen L.-C., Wu L.-L., Yu H.-P. (2021). Clinical implications of concentration of alveolar nitric oxide in asthmatic and non-asthmatic subacute cough. J. Breath. Res..

[19] Wang K., Stonham C., Rutherford C., Pavord I.D. (2023). Fractional exhaled nitric oxide (FeNO): The future of asthma care?. Br. J. Gen. Pr..

[20] Zeng G., Xu J., Zeng H., Wang C., Chen L., Yu H. (2024). Differential Clinical Significance of FENO200 and CANO in Asthma, Chronic Obstructive Pulmonary Disease (COPD), and Asthma-COPD Overlap (ACO). J. Asthma Allergy.

[21] Hara Y., Shinkai M., Kanoh S., Fujikura Y., Rubin B.K., Kawana A., Kaneko T. (2017). Arterial Carboxyhemoglobin Measurement Is Useful for Evaluating Pulmonary Inflammation in Subjects with Interstitial Lung Disease. Intern. Med..

[22] Cameli P., Bargagli E., Bergantini L., d’Alessandro M., Pieroni M., Fontana G.A., Sestini P., Refini R.M. (2020). Extended Exhaled Nitric Oxide Analysis in Interstitial Lung Diseases: A Systematic Review. Int. J. Mol. Sci..

[23] Raghu G., Remy-Jardin M., Myers J.L., Richeldi L., Ryerson C.J., Lederer D.J., Behr J., Cottin V., Danoff S.K., Morell F. (2018). Diagnosis of Idiopathic Pulmonary Fibrosis. An Official ATS/ERS/JRS/ALAT Clinical Practice Guideline. Am. J. Respir. Crit. Care Med..

[24] Collard H.R., Ryerson C.J., Corte T.J., Jenkins G., Kondoh Y., Lederer D.J., Lee J.S., Maher T.M., Wells A.U., Antoniou K.M. (2016). Acute Exacerbation of Idiopathic Pulmonary Fibrosis. An International Working Group Report. Am. J. Respir. Crit. Care Med..

[25] Dweik R.A., Boggs P.B., Erzurum S.C., Irvin C.G., Leigh M.W., Lundberg J.O., Olin A.-C., Plummer A.L., Taylor D.R. (2011). An official ATS clinical practice guideline: Interpretation of exhaled nitric oxide levels (FENO) for clinical applications. Am. J. Respir. Crit. Care Med..

[26] Horváth I., Barnes P.J., Loukides S., Sterk P.J., Högman M., Olin A.-C., Amann A., Antus B., Baraldi E., Bikov A. (2017). A European Respiratory Society technical standard: Exhaled biomarkers in lung disease. Eur. Respir. J..

[27] Spagnolo P., Ryerson C.J., Putman R., Oldham J., Salisbury M., Sverzellati N., Valenzuela C., Guler S., Jones S., Wijsenbeek M. (2021). Early diagnosis of fibrotic interstitial lung disease: Challenges and opportunities. Lancet Respir. Med..

[28] Brixey A.G., Oh A.S., Alsamarraie A., Chung J.H. (2024). Pictorial Review of Fibrotic Interstitial Lung Disease on High-Resolution CT Scan and Updated Classification. Chest.

[29] Miller M.R., Crapo R., Hankinson J., Brusasco V., Burgos F., Casaburi R., Coates A., Enright P., van der Grinten C.P.M., Gustafsson P. (2005). General considerations for lung function testing. Eur. Respir. J..

[30] Högman M., Bowerman C., Chavez L., Dressel H., Malinovschi A., Radtke T., Stanojevic S., Steenbruggen I., Turner S., Dinh-Xuan A.T. (2024). ERS technical standard: Global Lung Function Initiative reference values for exhaled nitric oxide fraction (F ENO50). Eur. Respir. J..

[31] Fortuna A.M., Balleza M., Calaf N., González M., Feixas T., Casan P. (2009). Determining the alveolar component of nitric oxide in exhaled air: Procedures and reference values for healthy persons. Arch. Bronconeumol..

[32] Deveci S.E., Deveci F., Açik Y., Ozan A.T. (2004). The measurement of exhaled carbon monoxide in healthy smokers and non-smokers. Respir. Med..

[33] Wuttge D.M., Bozovic G., Hesselstrand R., Aronsson D., Bjermer L., Scheja A., Tufvesson E. (2010). Increased alveolar nitric oxide in early systemic sclerosis. Clin. Exp. Rheumatol..

[34] Girgis R.E., Gugnani M.K., Abrams J., Mayes M.D. (2002). Partitioning of alveolar and conducting airway nitric oxide in scleroderma lung disease. Am. J. Respir. Crit. Care Med..

[35] Guilleminault L., Saint-Hilaire A., Favelle O., Caille A., Boissinot E., Henriet A.C., Diot P., Marchand-Adam S. (2013). Can exhaled nitric oxide differentiate causes of pulmonary fibrosis?. Respir. Med..

[36] Ricciardolo F.L.M., Sorbello V., Ciprandi G. (2015). A pathophysiological approach for FeNO: A biomarker for asthma. Allergol. Immunopathol..

[37] Cameli P., Bargagli E., Refini R.M., Pieroni M.G., Bennett D., Rottoli P. (2014). Exhaled nitric oxide in interstitial lung diseases. Respir. Physiol. Neurobiol..

[38] Cameli P., Barbagli E., Rottoli P. (2016). Exhaled nitric oxide is not increased in pulmonary sarcoidosis. Sarcoidosis Vasc. Diffus. Lung Dis..

[39] Chakraborty A., Mastalerz M., Ansari M., Schiller H.B., Staab-Weijnitz C.A. (2022). Emerging Roles of Airway Epithelial Cells in Idiopathic Pulmonary Fibrosis. Cells.

[40] Mackintosh J.A., Wells A.U., Cottin V., Nicholson A.G., Renzoni E.A. (2021). Interstitial pneumonia with autoimmune features: Challenges and controversies. Eur. Respir. Rev..

[41] Sen P., Khatri S.B., Tejwani V. (2023). Measuring exhaled nitric oxide when diagnosing and managing asthma. Cleve. Clin. J. Med..

[42] Johnson S.R., Bernstein E.J., Bolster M.B., Chung J.H., Danoff S.K., George M.D., Khanna D., Guyatt G., Mirza R.D., Aggarwal R. (2024). 2023 American College of Rheumatology (ACR)/American College of Chest Physicians (CHEST) Guideline for the Treatment of Interstitial Lung Disease in People with Systemic Autoimmune Rheumatic Diseases. Arthritis Rheumatol..

[43] Tiev K.P., Cabane J., Aubourg F., Kettaneh A., Ziani M., Mouthon L., Duong-Quy S., Fajac I., Guillevin L., Dinh-Xuan A.T. (2007). Severity of scleroderma lung disease is related to alveolar concentration of nitric oxide. Eur. Respir. J..

[44] Cameli P., Bergantini L., Salvini M., Refini R.M., Pieroni M., Bargagli E., Sestini P. (2019). Alveolar concentration of nitric oxide as a prognostic biomarker in idiopathic pulmonary fibrosis. Nitric Oxide.

[45] Cameli P., Bargagli E., Bergantini L., d’Alessandro M., Giugno B., Gentili F., Sestini P. (2021). Alveolar Nitric Oxide as a Biomarker of COVID-19 Lung Sequelae: A Pivotal Study. Antioxidants.

[46] Akira M., Kozuka T., Yamamoto S., Sakatani M. (2008). Computed tomography findings in acute exacerbation of idiopathic pulmonary fibrosis. Am. J. Respir. Crit. Care Med..

[47] Suzuki A., Kondoh Y., Brown K.K., Johkoh T., Kataoka K., Fukuoka J., Kimura T., Matsuda T., Yokoyama T., Fukihara J. (2020). Acute exacerbations of fibrotic interstitial lung diseases. Respirology.

[48] Ejazi M.A., Shameem M., Bhargava R., Ahmad Z., Akhtar J., Khan N.A., Alam M.M., Alam M.A., Adil Wafi C.G. (2018). Correlation of exhaled carbon monoxide level with disease severity in chronic obstruction pulmonary disease. Lung India.

[49] Trofor L., Miron R., Man M.A., Grosu I.-A., Trofor A.C. (2017). Correlations between lung function, exhaled carbon monoxide and „lung age” in smokers versus former smokers with COPD. Eur. Respir. J..

[50] Horvath I., Loukides S., Wodehouse T., Kharitonov S.A., Cole P.J., Barnes P.J. (1998). Increased levels of exhaled carbon monoxide in bronchiectasis: A new marker of oxidative stress. Thorax.

[51] Horváth I., Donnelly L.E., Kiss A., Paredi P., Kharitonov S.A., Barnes P.J. (1998). Raised levels of exhaled carbon monoxide are associated with an increased expression of heme oxygenase-1 in airway macrophages in asthma: A new marker of oxidative stress. Thorax.

[52] Vijayan V., Wagener F.A.D.T.G., Immenschuh S. (2018). The macrophage heme-heme oxygenase-1 system and its role in inflammation. Biochem. Pharmacol..

[53] Hull T.D., Agarwal A., George J.F. (2014). The mononuclear phagocyte system in homeostasis and disease: A role for heme oxygenase-1. Antioxid. Redox Signal.

[54] Steen E.H., Wang X., Balaji S., Butte M.J., Bollyky P.L., Keswani S.G. (2020). The Role of the Anti-Inflammatory Cytokine Interleukin-10 in Tissue Fibrosis. Adv. Wound Care.

[55] Lee T.-S., Chau L.-Y. (2002). Heme oxygenase-1 mediates the anti-inflammatory effect of interleukin-10 in mice. Nat. Med..

